# Suppression of JAK2/STAT3 Signaling Reduces End-to-End Arterial Anastomosis Induced Cell Proliferation in Common Carotid Arteries of Rats

**DOI:** 10.1371/journal.pone.0058730

**Published:** 2013-03-14

**Authors:** Jinbing Zhao, Meijuan Zhang, Wei Li, Xingfen Su, Lin Zhu, Chunhua Hang

**Affiliations:** 1 Department of Neurosurgery, Jinling Hospital, School of Medicine, Nanjing University, Nanjing, China; 2 Department of Neurosurgery, Nanjing Brain Hospital Affiliated to Nanjing Medical University, Nanjing, China; 3 Department of Neurology, Affiliated Drum Tower Hospital of Nanjing University medical school, Nanjing, China; Chang Gung University, Taiwan

## Abstract

**Background:**

JAK2/STAT3 pathway was reported to play an essential role in the neointima formation after vascular intima injury. However, little is known regarding this pathway to the whole layer injury after end-to-end arterial anastomosis (AA). Here, we investigated the role of JAK2/STAT3 pathway in common carotid arterial (CCA) anastomosis-induced cell proliferation, phenotypic change of vascular smooth muscle cells (VSMCs) and re-endothelialization.

**Methods:**

CCAs of adult male Wistar rats were resected at 3, 7, 14, and 30 days after end-to-end CCA anastomosis. Activation of JAK2/STAT3 pathway was detected by Western blotting and Immunofluorescence, and expression of proliferating cell nuclear antigen (PCNA) was detected by Q-PCR and Western blotting. Under the treatment with AG490 (a JAK2 inhibitor), protein levels of JAK2, STAT3 and PCNA, morphological changes of artery, phenotypic change of VSMCs, and re-endothelialization were measured by Western blotting, H&E, Q-PCR, and Evans blue staining respectively.

**Results:**

The protein levels of p-JAK2, p-STAT3, and PCNA were up-regulated, peaked on the 7^th^ day in the vessel wall after AA. AG490 down-regulated the levels of p-JAK2, p-STAT3, and PCNA on the 7^th^-day-group, resulting in reduced vessel wall proliferation on the 7^th^ and 14^th^ day after AA. Besides, AG490 switched the phenotypic change of VSMCs after AA representing inhibited mRNA levels of synthetic phase markers (osteopoitin and SMemb) and up-regulated contractile phase markers (ASMA, SM2 and SM22α). Furthermore, AG490 did not affect the re-endothelialization process on all indicated time points after AA (the 3^rd^, 7^th^, 14^th^, and 30^th^ day).

**Conclusion:**

Our study indicated that JAK2/STAT3 signaling pathway played an important role on cell proliferation of the injured vessel wall, and probably a promising target for the exploration of drugs increasing the patency or reducing the vascular narrowness after AA.

## Introduction

After the first human cerebral arterial anastomosis (AA) performed by Dr. Yasargil in 1967 connecting superficial temporal artery (STA) to middle cerebral artery (MCA), progressions in diagnostic methods and surgical techniques have led to a revival of cerebral revascularization procedures. End-to-end cerebral arterial anastomosis, as a conventional vascular reconstruction technique, is used widely in cerebral ischemia, aneurysms, and cranial base tumors [1]. However, the anastomosis procedure itself also induces vascular injury, which triggers the succedent repairing process including re-endothelialization, neointima formation, media vascular smooth muscle cells (VSMCs) proliferation, and adventitia regeneration [2], [3]. The repair process after vascular injury is associated with the up-regulation of adhesion molecules, recruitment of inflammatory cells and cytokines. Subsequently, the complicated repairing process leads to the alternation of the patency rate after revascularization, which would finally affect the clinical outcomes of patients.

The janus kinase/signal transducer and activator of transcription (JAK/STAT) is an important pathway responding to various kinds of cytokines and growth factors by transducing signals from cell surface to the nucleus in a wide variety of cell types [4]. Specifically, cytokines or growth factors firstly bind to their own receptors on the cell surface, which then recruits and activates the receptor-associated JAKs. Activated JAKs phosphorylate the tyrosine residues of the specific receptors, which could be recognized by the SH2 domains of STATs. STATs form the STAT homo- or hetero-dimers, and translocate into the nucleus, where they bind to specific DNA elements and modulate the expression of target genes [5], [6]. Inhibition of this pathway attenuated the neointima formation and cell proliferation after common carotid arteries (CCA) intima injury in previous studies [7], [8]. However, to our knowledge, there is still no report about the role of JAK2/STAT3 pathway after AA.

In this study, we investigated the activation characteristics of JAK2/STAT3 pathway in rats subjected to AA procedure. Furthermore, we studied the functional role of JAK2/STAT3 pathway in cell proliferation (including neointima, VSMCs, and adventitia), VSMCs phenotypic modulation, and re-endothelialization after AA with the hope of finding out the potential molecular mechanism underlying AA-induced cell proliferation and vascular restenosis.

## Materials and Methods

### Animals

Adult male Wistar rats (weight 300–350 g) were purchased from the Animal Center of the Academy of Military Medical Science (Beijing, China). Rats were housed in a humidified room with constant temperature (25°C), free access to water and food for at least one week before the experiment. All procedures were approved by the Nanjing University Animal Care and Use Committee (permit number: SYXK (jun) 2007-029, Nanjing, China) and in accordance with the Guide for the Care and Use of Laboratory Animals by the National Institute of Health (NIH Publication No. 85-23, revised 1996).

### End-to-end common carotid arterial anastomosis model

Using standard end-to-end arterial anastomosis technique as described previously [9], [10], CCA of both sides were cross sectioned successively and reconstructed by end-to-end anastomosis with 8 interrupted sutures (10–0 nylon). In brief, rats were fixed on supine position after intraperitoneal anesthesia with pentobarbital sodium (50 mg/kg). A middle incision from the larynx to the supraternal notch was performed, and CCA of both sides were exposed under blunt dissection of the muscles. Utilizing a LEICA operating microscope, CCA were transected after blood flow blocked by temporary clips. The advantitia near the edge of the arterial opening was trimmed. The initial sutures were placed approximately opposing each other at the 9:00 and 3:00 clock positions, and three more interrupted sutures for each side between the two initial sutures. After checking any obvious gaps between suture lines, distal and proximal clips were removed one by one. Small bleeding could be staunched easily by surrounding sterile gelatin sponge around the anastomosis and applying gentle pressure. Other than anesthesia, no anticoagulant, antiplatelet or vasodilator drugs were administered after the surgery.

### Experimental protocol

Anastomoses of bilateral CCAs of 70 rats were performed in this study. First, 8 rats (16 anastomoses) were randomly divided into 4 groups (n = 4 anastomoses per group): Sham-operated group (Sham), 3 arterial anastomsis (AA) groups for each time point (3, 7, and 14 consecutive days after AA). Sham-operated rats underwent the same operation except AA. At each time point, CCA of both sides were exposed to inspect vessel patency before perfusion with PBS (0.1 mol/L) under deep anesthesia. About 4-mm-long artery (2 mm proximal and 2 mm distal to the epicenter of anastomotic suture) was collected for Western blotting. The artery samples from groups with peak expression of p-STAT3 were used in immunofluorescence study (n = 3 anastomoses per group).

Further experiment involved the administration of a JAK2 inhibitor, AG490. AG490 (Cayman Chemical, MI, USA) was first dissolved in DMSO, and then in a harmless vector (25% pluronic F127 gel, Sigma, MO, USA) with the final concentration of 2 mg/ml. The 25% pluronic F127 gel will transform from liquid state at 4°C to jelly-like state at room temperature or above and was proved with high compatibility, so it is a perfect vector for employing AG490 surround the AA sites. After the AA procedures, gel containing AG490 or vehicle (DMSO) was applied around the AA sites (2 mg per site). Preliminary experiments showed there was no significant difference of all detected variables among Sham groups treated with nothing, DMSO, pluronic F127 gel, or AG490 respectively. According to the previous results, p-JAK2 and p-STAT3 expression peaked at 7 days after AA, so the 7^th^ day was set as the time point to inspect the effect of AG490 on JAK2/STAT3 pathway, PCNA expression and morphometrical changes. Four groups on the 7^th^ day: Sham, AA, AA+DMSO, and AA+AG490 (n = 8 anastomoses per group), were set in this part. Furthermore, the normal healing procedures after AA including vascular smooth muscle cells (VSMCs) phenotype change (Sham, AA+DMSO, and AA+AG490 groups on the 3^rd^, 7^th^, and 14^th^ day after AA) and re-endothelialization of intima (Sham, AA+DMSO, and AA+AG490 groups on the 3^rd^, 7^th^, 14^th^,and 30^th^ day after AA) were studied (n = 3 anastomoses per subgroup). Sample collection was the same as described above.

### Western blotting analysis

Total protein was extracted from sample cortex according to standard protocol, and adjusted to same concentration. 20 µg sample protein per lane was separated by 8–10% SDS-polyacrylamide gel electrophoresis (SDS-PAGE) and transferred to polyvinylidene-difluoride (PVDF) membrane. The membranes were incubated in 5% skimmed milk for 1 h, and overnight at 4 °C with primary antibodies (rabbit anti-rat JAK2, 1∶800 dilution, Santa Cruz; p-JAK2^Tyr1007/1008^, 1∶1000 dilution, Abcam; STAT3, 1∶800 dilution, Santa Cruz; p-STAT3^Tyr705^, 1∶1000 dilution, Cell Signaling Technology; PCNA, 1∶1000 dilution, Abcam; and β-actin 1∶2000 dilution, Santa Cruz). β-actin was used as a loading control. After washed with TBST, the membranes were incubated with secondary antibodies for 2 h at room temperature. Then the membranes exposed to the negative films to develop target bands after incubated with enhanced chemiluminescence (AmerSham, IL, USA). The intensities of bands were quantitated by NIH IMAGE-J software.

### Morphometry and immunofluorescent staining

Specific sample preparation included perfusion with 0.1 mol/L PBS (200 ml), followed by 200 ml of 4% neutral paraformaldehyde. About 2-mm-long artery (1 mm proximal and 1 mm distal to the epicenter of anastomotic suture) was dissected. Serial frozen tissue sections (10 µm thick, one section for every 100 µm-long artery, 20 sections totally) including the AA sutures were used for hematoxylin&eosin (H&E) and immunofluorescent (IF) staining. Morphometrical changes focused on the proliferation of all three arterial wall layers and were analyzed according to the results of H&E staining by assessing the outside radius (R_o_)/inside radius (R_i_) ratio. In brief, the radius of arteries could not be measured directly because of the oval shape of sample arteries. So, we measured the outside and inside circumference, and transformed it to radius using the following formula: R_o_/R_i_ = (outside circumference/2π)/(inside circumference/2π).

Procedures of IF staining of p-STAT3 were as follows: After incubated in 0.1% Triton x-100 for 20 min, and then in 5% fetal bovine serum for 60 min to quench non-specific binding, sections were incubated with primary antibody (rabbit anti rat p-STAT3, 1∶200 dilution) overnight at 4°C. Chromeo 546-labelled IgG (1∶1000 dilution, Abcam, MA, USA) was used to detect the immunoreactivity of p-STAT3. In addition, 4', 6-diamidino-2-phenylindole (DAPI) was used to show the cell nuclei.

### Quantitative real-time PCR

Total RNA was prepared using RNAiso Plus (TaKaRa Bio, Dalian, China) and according to the RNA extraction protocol. The concentration and purity of total RNA were determined by spectrophotometer (OD260/280 1.8–2.2) and 1% agarose gel electrophoresis. The primers were designed according to the coding domain sequence (CDS) of mRNA respectively (ASMA NM_031004.2, SM2 NM_001170600.1, SMemb NM_031520.1, SM22α NM_031549.2, Osteopontin NM_012881.2, PCNA NM_022381.3, and β-actin NM_031144.2. http://www.ncbi.nlm.nih.gov/nucleotide/for details). The primers were synthesized by Invitrogen Life Technologies (Shanghai, China). Sequences of primers were as follows:

PCNA (Proliferating cell nuclear antigen):

F: GGGCTGAAGATAATGCTGAT R: TTCTGGGATTCCAAGTTGCT

ASMA (Alpha smooth muscle actin):

F: ATGACCCAGATTATGTTTGAGACC R: CCAGAGTCCAGCACAATACCAG

SM2:

F: CAATGAGGCTTCTGTGCTGC R: ATGGGTAGGTGCTTGTAGGG

SM22α:

F: GAGGACTGTAATGGCTTTGG R: TTGACTGTCTGTGAACTCCCT

Osteopoitin:

F: CCCGATGCCACAGATGAG R: CTTCCCGTTGCTGTCCTG

SMemb:

F: ATTCAGTACCTTGCCCACG R: TCACAGTCTTCGCATTTCC

β-actin:

F: GACGTTGACATCCGTAAAGACC R: TGCTAGGAGCCAGGGCAGTA

Q-PCR reaction mixture was prepared according to the kit protocol (TaKaRa Bio, Dalian, China) and performed by ABI PRISM 7500 Real-Time PCR System (Applied Biosystems, CA, USA) in two steps (step 1: 95°C, 30 sec; step 2: 95°C, 5 sec and 60°C, 34 sec repeated 40 times). The relative mRNA expression were analyzed using the 2^−ΔΔCT^ method [11], and mRNA level in sham group was set as 1.

### Analysis of re-endothelialization

Re-endothelialization of the AA site was assessed by quantifying the Evans blue staining area by NIH IMAGE-J software. 6 ml 0.5% Evans blue dye (Sigma, MO, USA) was injected through the femoral vein 30 min before euthanasia. Following perfusion with 100% methanol through the left ventricle, sample CCA, including the AA site, was excised en bloc, dissected longitudinally, and photographed by operating microscope. Denuded/non-endothelialized area was defined as the area stained with Evans blue dye. However, it was difficult to measure the unregular distributed stained area around the AA site, so we transformed the photo into gray, measured the total pixels of 6-mm-long artery (including the AA site). The more denudation of artery, the larger stained area reflected, so the ratio (total pixels in AA groups/total pixels in Sham groups) was used to evaluate the level of re-endothelialization after AA.

### Statistical analysis

SPSS 15.0 was used for statistical analysis. All data were expressed as mean ± SE, and the statistical differences among different groups, different subgroups were assessed by one-way ANOVA with Fisher's LSD post-test. Statistical significance was set at 0.05.

## Results

### General parameters

All rats survived from the surgery of CCA anastomosis. There is no significant difference of body temperature, body weight, and mean arterial blood pressure detected in any experimental group (data not shown). The patency rate of CCA anastomosis in this study is 100% (no significant difference found between groups with or without AG490 treatment).

### Activation of JAK2/STAT3 signaling pathway in the vessel wall after AA

**Figure 1:**
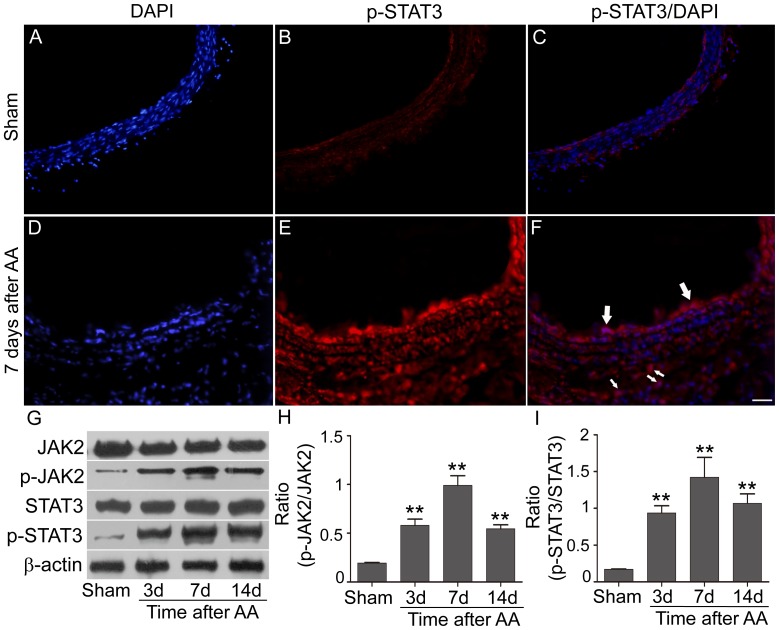
activation features of JAK2/STAT3 in the vessel wall after AA. **A–F**: p-STAT3 positive cells (red) in Sham group and AA group determined by immunofluorecence (Samples: 7 days after AA; Arrows indicated the typical positive cells. n = 3 anastomoses. Bar = 100 µm). **G–I**: Representative bands of target proteins in Sham group and AA groups at 3, 7, and 14 days after surgery (n = 4 anastomoses). The analysis showed the expression p-JAK2 and p-STAT3 increased and peaked at 7 days after the AA procedure (^**^ p<0.01 versus Sham group).

Western blotting was performed to determine the JAK2/STAT3 pathway activation after AA at indicated time points. Similar expression of JAK2 and STAT3 were shown in all detected groups (Fig. 1G). Both of p-JAK2 and p-STAT3 increased immediately after AA and peaked on the 7^th^ day (p-JAK2: 0.99±0.10 vs. 0.19±0.006 in Sham, p<0.01; p-STAT3: 1.42±0.27 vs. 0.17±0.004 in Sham, p<0.01), and still kept at a high level till to 14 days after AA (p<0.01; Fig. 1H, I). IF staining demonstrated p-STAT3 increased 7 days after AA and mainly located on the proliferated neointima and advantitia, while p-STAT3 was expressed at trace level in sham group (Fig. 1A–F).

### AG490 down-regulated JAK2/STAT3 expression in vessel wall and attenuated AA-induced cell proliferation

**Figure 2:**
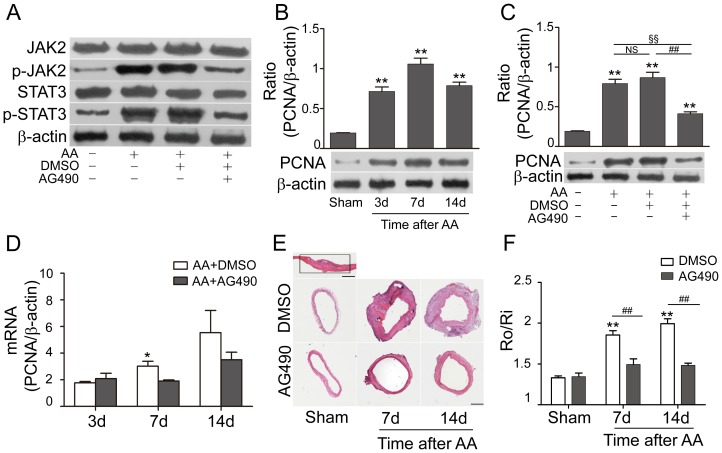
AG490 inhibited the up-regulation of p-JAK2, p-STAT3 and PCNA, and attenuated cell proliferation after AA. **A**: AG490 down-regulated the activation of JAK2/STAT3 in the AA+AG490 group on the 7^th^ day (n = 4 anastomoses). **B–D**: mRNA levels of PCNA were up-regulated after AA, and inhibited markedly after treating with AG490 on the 7^th^ day. Protein levels of PCNA were increased and peaked at the 7^th^ day after AA, and down-regulated in AA+AG490 group (n = 4 anastomoses). **E–F:** Morphometry analysis of CCA on the 7^th^ and 14^th^ day. Quantification analysis results were illustrated by the outside radius (R_o_)/inside radius (R_i_) ratio. Bar = 500 µm. n = 3 anastomoses. (^*^ p<0.05 versus AA+AG490 group (7^th^ day); ^**^ p<0.01 versus Sham group; ## p<0.01, §§ p<0.01; NS: no significant difference).

To further demonstrate the functional role of JAK2/STAT3 pathway after AA, a JAK2 inhibitor (AG490) was applied. Consistent with previous study [7], [8], phosphorylated JAK2 and STAT3 decreased in AG490-treated group on the 7^th^ day after AA (p-JAK2: 0.47±0.03 vs. 0.89±0.04 in AA+DMSO; p-STAT3: 0.49±0.06 vs. 1.09±0.11 in AA+DMSO, p<0.01, Fig. 2A).

PCNA, as a marker of cell proliferation, was detected in both mRNA and protein levels. Both mRNA levels and protein expression of PCNA were strengthened at 3, 7, 14 days after AA (p<0.01; Fig. 2B, D), and were inhibited by AG490 on the 7^th^ day (0.41±0.02 in AA+AG490 vs. 0.87±0.07 in AA+DMSO, p<0.01, Fig. 2C). Morphometric measurement of artery crossing sections near the AA site (the area marked with rectangle in Fig. 2E) showed the inhibitive effect of AG490 on cell proliferation. AG490 had no effect on the R_0_/R_i_ ratio of Sham groups treated with DMSO or AG490, but down-regulated the R_0_/R_i_ ratio between AA+AG490 group and AA+DMSO group on the 7^th^ and 14^th^ day after AA (1.49±0.07 vs. 1.85±0.05 on 7^th^ day, p<0.01; 1.48±0.03 vs. 1.99±0.06 on 14^th^ day, p<0.01; Fig. 2E–F).

### AG490 inhibited synthetic VSMCs proliferation in the AA sites

**Figure 3:**
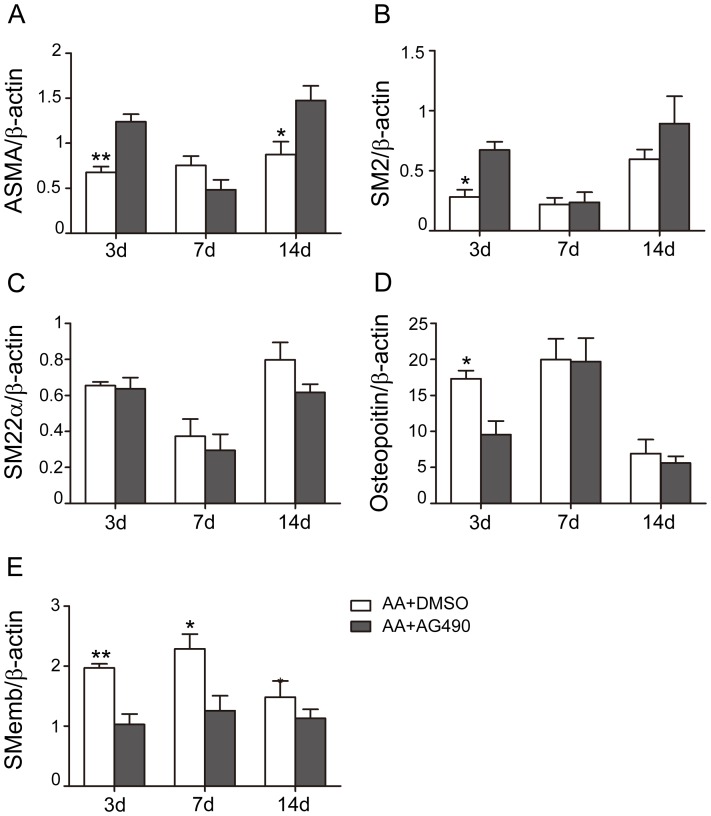
Phenotypic modulation of VSMCs by AG490 after AA procedure. The mRNA levels of ASMA, SM2, and SM22α was lowered after AA, while that of osteopoitin and SMemb were raised markedly and peaked at 7^th^ day after AA. At the 14^th^ day time point, expression of SM2 and SM22α began to increase but still lower than Sham group, and expression of osteopoitin and SMemb decreased to lower levels. AG490 down-regulated the osteopoitin and SMemb expression, and had a trend to increase the ASMA and SM2 mRNA levels. All the results were normalized to Sham. n = 4 anastomoses. (^*^ p<0.05, ^**^ p<0.01 versus AA+AG490 respectively).

Q-PCR detected the mRNA levels of different phenotypic markers of VSMCs, including ASMA, SM2, and SM22α for contractile phenotype, osteopoitin and SMemb for synthetic phenotype. Level of ASMA was significantly lowered on the 3^rd^ day after AA (0.68±0.06 vs. 1±0.04 in Sham, p<0.05), and then gradually increased to nearly normal level (0.87±0.14 on the 14^th^ day after AA, p>0.05). mRNA levels of SM2 and SM22α decreased in AA groups on different time points, and reached the lowest levels on the 7^th^ day after AA (SM2: 0.22±0.05 vs. 1±0.003 in Sham, p<0.01; SM22α: 0.37±0.10 vs. 1±0.06 in Sham, p<0.01, Fig. 3B, C). However, mRNA of osteopoitin and SMemb were up-regulated significantly after AA, and peaked on the 7^th^ day (Osteopoitin: 19.98±2.88 vs. 1±0.01 in Sham, p<0.01; SMemb: 2.29±0.25 vs.1±0.02 in Sham, p<0.01; Fig. 3D, E). AG490 inhibited the proliferation of synthetic phenotypic VSMCs (Osteopoitin: 9.55±1.89 vs. 17.32±1.12 in AA+DMSO, p<0.05; SMemb: 1.03±0.17 vs. 1.97±0.07 in AA+DMSO, p<0.01, Fig. 3D, E), and had a trend to enhance that of contractile phenotypic VSMCs.

### AG490 had no effect on the normal re-endothelialization progress after AA

**Figure 4:**
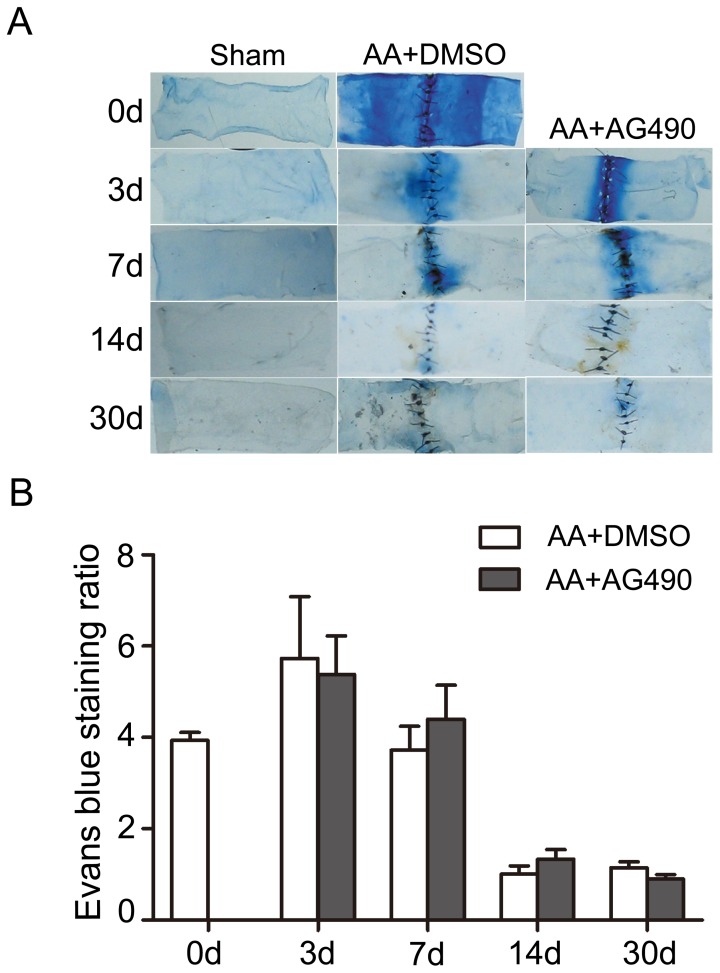
Re-endothelialization assessment on AA site at different experimental time points. Quantification analysis of the blue staining area demonstrated heavy staining in and around the AA sites, which peaked at the 3^rd^ day, and regressed to normal level at the 30^th^ day after AA. No statistic difference was found between the AA+DMSO and AA+AG490 groups at all indicated time points. n = 3 anastomoses.

Evans blue staining was performed to detect the intactness of endothelia after AA. The normal repair process of the AA-induced endothelia injury was showed in Fig. 4, which showed the Evans blue staining ratio rise after AA, peaked at the 3^rd^ day and fell off gradually to normal level after 30 days recovery. Administration of AG490 didn't affect the re-endothelialization process after AA when compared with vehicle treated groups.

## Discussion

The main findings of this experiment are: (1) JAK2/STAT3 signaling pathway was activated in the vessel wall after AA, and AG490 treatment down-regulated its activation; (2) AG490 attenuated AA-induced cell proliferation; (3) AG490 induced phenotypic switching of VSMCs by inhibiting the proliferation of synthetic phenotypic VSMCs and enhancing that of the contractile phenotypic ones; (4) Re-endothelialization after AA was not affected by AG490. Our study indicated that JAK2/STAT3 signaling pathway played an important role on cell proliferation of the injured vessel wall, and was probably a promising target for the exploration of drugs increasing the patency or reducing the vascular narrowness after AA.

End-to-end arterial anastomosis is a conventional arterial anastomosis technique used in clinic treatment. The reconstruction procedure itself induces vascular injury and the succedent repair process including re-endothelialization, neointima formation, VSMCs phenotype change and proliferation and adventitia regeneration. In the past decades, a variety of researches about vascular injury have been designed using different models. The rat CCA balloon injury was first described by Clowes and his colleagues in 1983 [12], and became one of the most highly characterized and used models for investigating the morphological and molecular response to experimental arterial injury. Flexible or spring wire induced arterial injury in mice was basically developed from the rat CCA balloon injury with various modifications [13], [14]. Besides, cuff placement around femoral artery, iron chloride or homocysteine induced injury were also used in some researches [15], [16], [17]. However, most basic researches about vascular injury are only focused on the neointima formation after the endothelium denuded, not the whole layers of artery; while CCA anastomosis model was designed to improve anastomosis techniques [18], [19]. Whatever, few researches focused on the AA-induced cytokine signaling pathways, which play important roles in the repairing process after vascular injury. That's the purpose of this experiment, and the reason why we chose CCA arterial anastomosis model.

Inflammatory response is an essential component of the repairing process after vascular injury, which is associated with the up-regulation of adhesion molecules, recruitment of inflammatory cells, growth factors, and cytokines. Recent researches found persistent up-regulation of cytokines played an important role in the development of many vascular dysfunction and vascular diseases, including atherosclerosis [20], aneurysm [21], hypertension [22] and varicose vein [23]. A variety of cytokines, including most interferons (IFNs), interleukins (ILs), and colony stimulating factors (CSFs) mediate their effects through the JAK/STAT pathway. So, in this experiment, we focused to investigate the activation of JAK2/STAT3 pathway after AA and whether the inhibition of this pathway could do benefit for the artery reconstruction. In several previous researches on vascular injury, STAT3 phosphorylation was enhanced with increased migration of VSMCs from media layer into neointima, and negative regulation with adenoviral vector encoding dnSTAT3 reduced the cell migration and neointima formation [8], [24]. Torella et.al also demonstrated that the inhibitive effect of fludarabine on neointimal hyperplasia was specifically associated with the down-regulation of STAT1 [25]. Besides, Kundumani-Sridharan et.al found blockade of STAT5B activation after balloon injury also resulted in reduced neointima formation [26]. All of STAT1, STAT3, and STAT5B could be activated by JAK2, whose phosphorylation was detected in the media and neointimal VSMCs after balloon injury; and treatment with a JAK2 inhibitor (AG490) could attenuate the neointimal proliferation [7].

However, most previous researches about JAK/STAT in vascular remodeling concentrated on the neointima formation, migration of VSMCs, there was still no consentaneous conclusion about its role in neointima formation [27], [28], and no report illustrated the importance of JAK/STAT pathway in adventitia. In the present study, we did found the activation of JAK2/STAT3 pathway in the whole vessel wall, showing p-STAT3 positive cells in both neointima and adventitia. Application of AG490 inhibited the activation of the pathway, which resulted in less cell proliferation and preventing vascular narrowness during the remodeling after AA.

An essential factor in the pathogenesis of restenosis after vascular injury is the VSMC migration from media into intima and its proliferation in intima, which is associated with the phenotypic change. Previous studies have demonstrated the phenotypic change of VSMC during different vascular diseases. Oxidized phospholipids within atherosclerotic lesions, could induce phenotypic switching of VSMCs by suppressing the expression of ASMA and SM-MHC [29]. And proliferative phenotypic VSMCs contributed to the plaque growth and formation of fibrotic cap in atherosclerosis. Abnormal VSMCs induced the expression of matrix metalloproteinase 9 (MMP-9) mediating elastolysis in a mouse model of Marfan syndrome [30]. During this study, we used the following five markers to demonstrate the switch of VSMCs: ASMA, SM2, SM22α, osteopoitin, and SMemb. ASMA and SM22α are two well-known VSMCs differentiation markers [31]. SM2 and SMemb are two isoforms of smooth muscle myosin heavy chain (MHC). Most VSMCs were SM2+/SMemb- cells in the media layer of intact artery, while SMemb+ cells appeared in the media and occupied almost the entire neointima after experimental carotid artery injury [7], [32], suggesting SM2 and SMemb could be markers for differentiated and de-differentiated VSMCs respectively. In addition to SMemb, osteopoitin is also a marker for the proliferative synthetic VSMCs [31], [33].

JAK/STAT pathway, as a main pathway downstream of cytokines and growth factors receptors, has been found activated in VSMCs under the stimulation of different factors, including IL-6 [34], platelet-derived growth factor-BB [35], and angiotensin II [36]. It suggested the importance of JAK/STAT in VSMCs, and AG490 could inhibit the VSMCs motility and proliferation by blockading the activation of JAK2/STAT1/STAT3 [35], [36]. However, no report has investigated the relationship between AG490 and the phenotype switching of VSMCs *in vivo* or *in vitro*. Here, our data implied that AG490 attenuated the cell proliferation after AA probably by switching the proliferative phenotype back to the quiescent state of VSMCs.

The endothelia repair progressed in stages after vascular injury [37]. Stemerman et.al investigated the intimal healing after aorta endothelium denudation by balloon injury, which included re-endothelialization and intimal thickening [37]. The repair process began with the aggregation of single platelets and leykocytes in the de-endothelialized surface, and the slowly migration of endothelial cells (ECs) from the intact endothelium to the injury surface. The regions not covered with ECs were covered by VSMCs origin cells, which were permeable to Evans blue conjuncated proteins. This made Evan blue to be a useful tool to detect the de-endothelialized area. Previous researches in cell cultures have revealed the activation of JAK/STAT signaling in ECs under the stimulation of cytokines, growth factors, or abnormal conditions, including hypoxia and loss of shear stress. Dudley and colleagues reported the activation of JAK2/STAT5 in vascular ECs under low oxygen condition, and the activation may relate to the angiogenesis of neoplasm and retinopathy [38]. Another paper from Ni and his group found the normal constant shear stress could down-regulate the IL-6-induced phosphorylation of JAK2/STAT3 in ECs and subsequent ECs proliferation [39]. Neria, for the first time, reveled blockade of JAK2 with AG490 in ECs cultures could significantly protect the ECs under the detached condition or serum deprivation, and increase the growth capability in reseeding [40]. However, there is still no *in vivo* study about the effect of AG490 on re-endothelialization after vascular injury. Our results revealed that the Evans blue-stained area in both vehicle- and AG490-treated groups reduced to almost normal level at 14 days after AA, and we didn't find AG490 accelerate the repairing process of endothelium after AA.

In conclusion, to our knowledge, we are the first to demonstrate the activation pattern of JAK2/STAT3 pathway in common carotid arterial anastomosis model. And the blockade effect from AG490 induced the down-regulation of cell proliferation after AA. Besides, AG490 could switch back the phenotypic change of VSMCs by inhibiting the synthetic phase and up-regulating the contractile phase, which was much more important for re-obtaining the contractile ability. Moreover, the AG490 treatment had no detrimental effect on the re-endothelialization of artery. These results suggest a potential therapeutic role of AG490 in the vascular repairing process after AA.
